# Interaction between Polymorphisms in Pre-MiRNA Genes and Cooking Oil Fume Exposure on the Risk of Lung Cancer in Chinese Non-Smoking Female Population

**DOI:** 10.1371/journal.pone.0128572

**Published:** 2015-06-17

**Authors:** Zhihua Yin, Zhigang Cui, Peng Guan, Xuelian Li, Wei Wu, Yangwu Ren, Qincheng He, Baosen Zhou

**Affiliations:** 1 Department of Epidemiology, School of Public Health, China Medical University, Shenyang 110013, PR China; 2 Key Laboratory of Cancer Etiology and Intervention, University of Liaoning Province, Shenyang 110013, PR China; 3 China Medical University, Shenyang 110013, PR China; Sapporo Medical University, JAPAN

## Abstract

**Background:**

Both genetic polymorphisms and environmental risk factors play important roles in the development of human chronic diseases including lung cancer. This is the first case-control study of interaction between polymorphisms in pre-miRNA genes and cooking oil fume exposure on the risk of lung cancer.

**Methods:**

A hospital-based case-control study of 258 cases and 310 controls was conducted. Six polymorphisms in miRNAs were determined by Taqman allelic discrimination method. The gene-environment interactions were assessed on both additive and multiplicative scale. The statistical analyses were performed mostly with SPSS.

**Results:**

The combination of the risk genotypes of five miRNA SNPs (miR-146a rs2910164, miR-196a2 rs11614913, miR-608 rs4919510, miR-27a rs895819 and miR-423 rs6505162) with risk factor (cooking oil fume exposure) contributed to a significantly higher risk of lung cancer, and the corresponding ORs (95% confidence intervals) were 1.91(1.04-3.52), 1.94 (1.16-3.25), 2.06 (1.22-3.49), 1.76 (1.03-2.98) and 2.13 (1.29-3.51). The individuals with both risk genotypes of miRNA SNPs and exposure to risk factor (cooking oil fumes) were in a higher risk of lung cancer than persons with only one of the two risk factors (ORs were 1.91, 1.05 and 1.41 for miR-146a rs2910164, ORs were 1.94, 1.23 and 1.34 for miR-196a2 rs11614913, ORs were 2.06, 1.41 and 1.68 for miR-608 rs4919510, ORs were 1.76, 0.82 and 1.07 for miR-27a rs895819, and ORs were 2.13, 1.15 and 1.02 for miR-423 rs6505162, respectively). All the measures of biological interaction indicate that there were not indeed biological interactions between the six SNPs of miRNAs and exposure to cooking oil fumes on an additive scale. Logistic models suggested that the gene-environment interactions were not statistically significant on a multiplicative scale.

**Conclusions:**

The interactions between miRNA SNPs and cooking oil fume exposure suggested by ORs of different combination were not statistically significant.

## Background

In China, lung cancer has become the leading cause of cancer-related deaths for both men and women [1]. The incidence and death rate of lung cancer in Chinese urban populations have reached the number one among malignant tumors [2]. Smoking is the main risk factor for lung cancer; however, it is estimated that 15% male and 53% female of lung cancer patients are nonsmokers [3], which suggests that other risk factors have also been implicated in the etiology of lung cancer. Cigarette smoking cannot fully explain the epidemiologic characteristics of lung cancer in Chinese women, so it’s really significant to explore other important risk factors for lung cancer in Chinese non-smoking females in order to control the effect of cigarette smoke.

Exposure to environmental risk factors play important roles in the development of lung cancer, but in the same environment people differ in their susceptibility to cancer. Some of these differences are ascribed to the concept that genetic susceptibility modifies the effects of environmental exposures [4].

Molecular epidemiologic studies showed that there were hundreds of genes involved in lung carcinogenesis [5]. In recent years, emerging evidence supports an extraordinary role for microRNAs (miRNAs) in the pathogenesis of human lung cancer. MiRNAs are classes of non-coding RNAs, approximately 20 nucleotides in length, which are considered to regulate expression by binding to cis-regulatory regions of 3’-UTR regions of target genes [6]. MiRNAs have been implicated in crucial biological processes, including development, differentiation, apoptosis, proliferation, and the development of most diseases including cancer [7]. The study showed that more than 50% of miRNA genes locate in cancer-related genomic regions or in fragile sites, suggesting that miRNAs may play important roles in cancer development [8]. Single nucleotide polymorphisms (SNPs) in the seed region or the precursor stem-loop of miRNA are reported to significantly affect the production or processing of miRNA [9–10]. These lines of evidence suggest that miRNA SNPs might affect the expression or function of their host miRNAs. Now the researchers conclude that SNP or mutations in miRNA sequence may alter expression, maturation and/or target selection of miRNA, and consequently contribute to modify cancer risk. In the last few years, the association between miRNA SNPs and the risk of cancer has become the hot topic in cancer etiology research.

There are studies on the relationship between miRNA SNPs and lung cancer risks. For example, Tian et al. found that the rs11614913 SNP in miR-196a2 was associated with the susceptibility of lung cancer, however, no significant effects were observed on the association for the other three SNPs (rs2910164, rs2292832, and rs3746444) located at the pre-miRNA regions of miR-146a, miR-149, miR-196a2, and miR-499[11]. The recent study in Korean suggested that the rs2910164C>G in pre-miR-146a may contribute to genetic susceptibility to lung cancer [12]. A meta-analysis showed that lung cancer risk was associated with SNP rs11614913 but not with SNP rs3746444 [13].

We have studied the effect of the common SNPs in selected six miRNAs on the susceptibility of lung cancer in non-smoking females (miR-146a rs2910164, miR-196a2 rs11614913, miR-30c-1 rs928508, miR-608 rs4919510, miR-27a rs895819, and miR-423 rs6505162) and found the significantly associated miRNA SNPs (the manuscript has been submitted). However, among people carrying same genotype of SNPs, some of them develop cancer and others not. In the same environment, people differ in their susceptibility to cancer. Some of these differences may due to modification of genetic factors on the effects of environmental risk factors. Exploring gene-environment interaction, which combines the genetic susceptibility and environmental exposures together, may be the good way to understand the etiology of cancer. The interaction of the miRNA SNPs and environmental risk factors on lung cancer susceptibility has not been reported so far.

Cooking oil fume exposure is suggested to be a risk factor of lung cancer in previous studies. Our previous reports showed that household exposure to cooking oil fume was associated with the risk of non-smoking female lung cancer [14–18]. A study in a high incidence population in North East India found that the interaction of XRCC1Gln/Gln genotype with exposure of exposure of cooking oil fumes was significantly associated with increased risk for lung cancer [19]. There are some reviews on the risk factors of lung cancer concluded the significant association between cooking oil fume exposure and lung cancer risk [20–21].

In the previous general interaction analyses, logistic regression has been commonly. However, in logistic or Cox regression model, the regression coefficient of the product term estimates the interaction on a multiplicative scale while statistical significance indicates the departure from multiplicativity. Rothman argues that when biologic interaction is examined, we need to focus on interaction as departure from additivity rather than departure from multiplicativity [22]. In this paper, we calculate both the additive interaction and the multiplicative interaction. The results can be used as reference by epidemiologists to assess the biological interaction between factors.

To our knowledge, this is the first and comprehensive case-control study of lung cancer to evaluate the interaction between the SNPs in pre-miRNAs and exposure to environmental risk factor in the development of lung cancer. In this study, we describe a case-control study of lung cancer in non-smoking female population in Shenyang, northeast China to study the interaction between miRNA SNPs and cooking oil fume exposure.

## Materials and Methods

### Ethics Statement

The study was approved by the Institutional Review Board of China Medical University, Shenyang, China. All patients provided written informed consent.

### Study subjects and data collection

This is an ongoing molecular epidemiologic study of lung cancer in Shenyang City, located in northeast China. Each subject was interviewed to collect demographic data and environmental factors, as well as 5ml venous blood to detect SNP. Environmental factors include passive smoking, cooking oil fume exposure, fuel smoke exposure, family history of cancer, occupational exposure and dietary habit, which were obtained for each case and control by trained interviewers. Individual with a total of 100 cigarettes in his lifetime was defined as a smoker, otherwise he was considered as a non-smoker.

In this hospital-based case-control study, study subjects included 258 cases and 310 matched controls for age and gender, consecutively were enrolled to the study during July 2006 and December 2012. The inclusion of case group: (1) newly diagnosed lung cancer patients, (2) histologically confirmed lung cancer by experienced pathologists, (3) never receiving chemotherapy or radiotherapy, (4) nonsmoking female patients, (5) capability for undergoing a 1.5 hour interview, (6) with the complete information of environmental exposure. The exclusion criteria included previous cancer or metastasized cancer from a different tumor. During the same period, the controls consisted of healthy people visiting other than the cases or other cancer patients were recruited from medical examination centers in the same hospital, excluding those with neoplasm and respiratory diseases. Controls were all non-smoking females and frequency matched to cases on age (±5 years). Participants were unrelated ethnic Han Chinese women.

In our previous published papers, cooking oil fume exposure was found to be significantly associated with the risk of lung cancer in non-smoking females [14–18], so in the present study we analyze the interaction between SNPs and cooking oil fume exposure. For cooking oil fume exposure, participants were asked about the frequency of cooking and types of oils. Subjects were also asked “How often did the air in your kitchen become filled with oily ‘smoke’ during cooking?” For each of these questions, there were four possible responses ranging from “never”, “seldom”, “sometimes”, to “frequently”. Exposure for cooking oil fume was categorized as an indicator variable equal to 1 if participants reported frequently or sometimes, and equal to 0 otherwise.

### SNP genotyping

Genomic DNA samples were isolated using Phenol-chloroform Method. Genotyping of the SNPs was done on an Applied Biosystems 7500 FAST Real-Time PCR System (Foster City, CA, USA) using Taqman allelic discrimination (Applied Biosystems, Foster City, CA) with a commercially available primer probe set (assay ID C_15946974_10 for rs2910164, C_31185852_10 for rs11614913, C_1766455_10 for rs928508, C_2826025_10 for rs4919510, C_3056952_20 for rs895819 and C___11613678_10 for rs6505162). Quality controls included that appropriate negative controls were included in each run, and 10% of patients were randomly selected to be genotyped twice by different persons and the results were found to be concordant for all of the masked duplicate sets.

### Statistical analysis

Unconditional logistic regression analysis was performed to calculate the odds ratios (OR) and their 95% confidence intervals (CI) for evaluating the associations between combination or interaction of SNPs and environment factors with lung cancer or lung adenocarcinoma risks. The gene-environment interaction was assessed by crossover analysis (additive interaction) and logistic regression models (multiplicative interaction). In these analyses, the combinations of protective genotypes of each SNP and absence of cooking oil fume exposure were considered as reference group. We use regression coefficients and covariance matrix of logistic model in SPSS software, and then introduce an Excel spreadsheet set up by Tomas Andersson to calculate the indices of interaction on an additive scale and the corresponding confidence intervals [23]. Three measures of biological interaction: the relative excess risk due to interaction (RERI), the attributable proportion due to interaction (AP), and the synergy index (S); as well as their 95%CIs were calculated based on the three relative risk estimates and the corresponding covariance matrix from a logistic regression model. Most of the statistical analyses were performed using SPSS 20.0 software, except for the measures of additive interactions as specified above. All of the tests were two-sided and statistical significance was defined as P<0.05.

## Results

There are 258 cases and 310 controls in the present analyses. All individuals are nonsmoking females. Of the 258 lung cancer cases, 194 (75.19%) patients were adenocarcinoma, 34 (13.18%) were squamous cells carcinoma and 30 (11.63%) were other types.

Analyses of environmental impact factors suggested that there was a significant association between lung cancer risk with cooking oil fume exposure (OR = 1.52, 95%CI = 1.06–2.17, P = 0.023) but not with other environmental risk factors (data not shown). In our previous studies and the present analyses, CG or GG genotype of rs2910164, TT genotype of rs11614913, AG or GG genotype of rs928508, GG genotype of rs4919510, TC or CC genotype of rs895819, CA or AA genotype of rs6505162 were suggested to be protective genotype(s), so the combinations of these genotypes and non-exposure to cooking oil fume were defined as reference groups in the interaction analyses.

Table 1 showed the comprehensive combination of six SNPs in miRNAs and cooking oil fume exposure on susceptibility of lung cancer and lung adenocarcinoma in Chinese non-smoking female population. The subjects carrying miR-196a2 rs11614913 TC genotype and exposure to cooking oil fume had a 2.08-fold risk of lung cancer compared with the combination of TT genotype and non-exposure to cooking oil fume (95%CI = 1.18–3.65). Individuals with miR-608 rs4919510 GC genotype and exposure to cooking oil fume had a 2.33-fold increased risk of cancer compared with the combination of GG genotype and non-exposure to cooking oil fume (95%CI = 1.29–4.23). The combination of miR-423 rs6505162 CC genotype and cooking oil fume exposure was significantly associated with lung cancer risk compared with the combination of AA genotype and non-exposure to cooking oil fume (OR = 3.14, 95%CI = 1.02–9.62). The similar results were showed in lung adenocarcinoma. Although in other SNPs, the results were not significant, it could be suggested that the combinations of risk genotypes and cooking oil fume exposure might be associated with the increased risks of lung cancer. In addition, the combination of SNPs in miRNAs and cooking oil exposure on susceptibility of lung cancer and lung adenocarcinoma under other comparisons were presented in S1 Table.

**Table 1:** Combination of SNPs in miRNAs and cooking oil exposure on susceptibility of lung cancer and lung adenocarcinoma in Chinese non-smoking female population.

SNP	oil	No of controls (%)	lung cancer			lung adenocarcinoma		
			No (%)	OR [95%CI]	P value	No (%)	OR [95%CI]	P value
rs2910164								
GG	Non-exposure	43 (13.9)	29 (11.2)	1.00 (ref)		23 (11.9)	1.00 (ref)	
CG	Non-exposure	119 (38.4)	88 (34.1)	1.10(0.64–1.89)	0.741	64 (33.0)	1.01(0.56–1.82)	0.986
CC	Non-exposure	66 (21.3)	50 (19.4)	1.12(0.62–2.04)	0.703	38 (19.6)	1.08(0.57–2.05)	0.823
GG	Exposure	20 (6.5)	16 (6.2)	1.19(0.53–2.66)	0.679	12 (6.2)	1.12(0.47–2.70)	0.797
CG	Exposure	41 (13.2)	46 (17.8)	1.66(0.89–3.13)	0.114	33 (17.0)	1.51(0.76–2.98)	0.241
CC	Exposure	21 (6.8)	29 (11.2)	2.05(0.98–4.26)	0.055	24 (12.4)	2.14(0.99–4.63)	0.055
rs11614913								
TT	Non-exposure	71 (22.9)	45 (17.4)	1.00 (ref)		32 (16.5)	1.00 (ref)	
TC	Non-exposure	112 (36.1)	91 (35.3)	1.28(0.81–2.04)	0.295	69 (35.6)	1.37(0.82–2.29)	0.233
CC	Non-exposure	45 (14.5)	31 (12.0)	1.09(0.60–1.96)	0.782	24 (12.4)	1.18(0.62–2.26)	0.611
TT	Exposure	26 (8.4)	22 (8.5)	1.34(0.68–2.63)	0.405	14 (7.2)	1.20(0.55–2.59)	0.652
TC	Exposure	38 (12.3)	50 (19.4)	**2.08(1.18–3.65)** *	**0.011**	42 (21.6)	**2.45(1.34–4.49)** *	**0.004**
CC	Exposure	18 (5.8)	19 (7.4)	1.67(0.79–3.51)	0.180	13 (6.7)	1.60(0.70–3.66)	0.263
rs928508								
GG	Non-exposure	47 (15.2)	41 (15.9)	1.00 (ref)		32 (16.5)	1.00 (ref)	
AG	Non-exposure	113 (36.5)	82 (31.8)	0.83(0.50–1.38)	0.476	59 (30.4)	0.77(0.44–1.33)	0.343
AA	Non-exposure	68 (21.9)	44 (17.1)	0.74(0.42–1.31)	0.300	34 (17.5)	0.73(0.40–1.35)	0.321
GG	Exposure	12 (3.9)	16 (6.2)	1.53(0.65–3.60)	0.332	14 (7.2)	1.71(0.70–4.18)	0.237
AG	Exposure	41 (13.2)	45 (17.4)	1.26(0.69–2.28)	0.450	31 (16.0)	1.11(0.58–2.12)	0.751
AA	Exposure	29 (9.4)	30 (11.6)	1.19(0.61–2.30)	0.613	24 (12.4)	1.22(0.60–2.46)	0.586
rs4919510								
GG	Non-exposure	70 (22.6)	40 (15.5)	1.00 (ref)		31 (16.0)	1.00 (ref)	
GC	Non-exposure	119 (38.4)	96 (37.2)	1.41(0.88–2.26)	0.153	69 (35.6)	1.31(0.78–2.20)	0.306
CC	Non-exposure	39 (12.8)	31 (12.0)	1.39(0.76–2.56)	0.290	25 (12.9)	1.45(0.75–2.79)	0.270
GG	Exposure	26 (8.4)	25 (9.7)	1.68(0.86–3.30)	0.129	15 (7.7)	1.30(0.61–2.80)	0.497
GC	Exposure	33 (10.6)	44 (17.1)	**2.33(1.29–4.23)** *	**0.005**	36 (18.6)	**2.46(1.31–4.64)** *	**0.005**
CC	Exposure	23 (7.4)	22 (8.5)	1.67(0.83–3.38)	0.150	18 (9.3)	1.77(0.84–3.73)	0.136
rs895819								
CC	Non-exposure	9 (2.9)	12 (4.7)	1.00 (ref)		9 (4.6)	1.00 (ref)	
TC	Non-exposure	86 (27.7)	66 (25.6)	0.58(0.23–1.45)	0.240	42 (21.6)	0.49(0.18–1.32)	0.158
TT	Non-exposure	133 (42.9)	89 (34.5)	0.50(0.20–1.24)	0.135	74 (38.1)	0.56(0.21–1.46)	0.235
CC	Exposure	9 (2.9)	5 (1.9)	0.42(0.10–1.68)	0.218	4 (2.1)	0.44(0.10–1.99)	0.288
TC	Exposure	39 (2.9)	37 (14.3)	0.71(0.27–1.89)	0.494	25 (12.9)	0.64(0.22–1.84)	0.407
TT	Exposure	34 (11.0)	49 (19.0)	1.08(0.41–2.85)	0.875	40 (20.6)	1.18(0.42–3.30)	0.757
rs6505162								
AA	Non-exposure	11 (3.5)	5 (1.9)	1.00 (ref)		4 (2.1)	1.00 (ref)	
CA	Non-exposure	74 (23.9)	52 (20.2)	1.55(0.51–4.72)	0.444	39 (20.1)	1.45(0.43–4.85)	0.547
CC	Non-exposure	143 (46.1)	110 (42.6)	1.69(0.57–5.01)	0.342	82 (42.3)	1.58(0.49–5.11)	0.448
AA	Exposure	3 (1.0)	2 (0.8)	1.47(0.18–11.72)	0.718	2 (1.0)	1.83(0.22–15.33)	0.576
CA	Exposure	32 (10.3)	22 (8.5)	1.51(0.46–4.96)	0.495	18 (9.3)	1.55(0.43–5.57)	0.505
CC	Exposure	47 (15.2)	67 (26.0)	**3.14(1.02–9.62)** *	**0.046**	49 (25.3)	2.87(0.85–9.64)	0.089

*: statistically significant results.

Table 2 showed the crossover analyses results about the interaction between SNPs in miRNAs and cooking oil fume exposure on lung cancer susceptibility in Chinese non-smoking female population. For miR-146a rs2910164 polymorphism, the individuals with CC genotype (risk genotype) and exposure to cooking oil fumes (risk factor) were more likely to develop lung cancer comparing to those with CG/GG genotype and non-exposure to cooking oil fume (OR = 1.91, 95% CI = 1.04–3.52, P = 0.037). The similar statistically significant results were also found in miR-196a2 rs11614913, miR-608 rs4919510, miR-27a rs895819 and miR-423 rs6505162 polymorphisms, that was the combination of the risk genotypes of these miRNA SNPs with risk factor of cooking oil fume exposure contributed to a significantly higher risk of lung cancer, and the corresponding ORs (95%CIs) were 1.94 (1.16–3.25), 2.06 (1.22–3.49), 1.76 (1.03–2.98) and 2.13 (1.29–3.51). In table 2, it could be found that people both carrying risk genotypes of miRNA SNPs and exposure to risk factor (cooking oil fumes) were in a higher risk of lung cancer than persons with only one of the two risk factors (ORs were 1.91, 1.05 and 1.41 for miR-146a rs2910164, ORs were 1.94, 1.23 and 1.34 for miR-196a2 rs11614913, ORs were 2.06, 1.41 and 1.68 for miR-608 rs4919510, ORs were 1.76, 0.82 and 1.07 for miR-27a rs895819, and ORs were 2.13, 1.15 and 1.02 for miR-423 rs6505162, respectively), which suggested that there may be interaction between these SNPs and cooking oil fume exposure, so we further explored the interaction on an additive scale and a multiplicative scale by quantitative and statistically significant analyses.

**Table 2:** Interaction between SNPs in miRNAs and cooking oil exposure on lung cancer susceptibility in Chinese non-smoking female population.

SNP	oil	No of controls (%)	No of cases (%)	OR (95%CI)	P value
rs2910164					
CG+GG	Non-exposure	162 (52.3)	117 (45.3)	1.00 (ref)	—
CC	Non-exposure	66 (21.3)	50 (19.4)	1.05 (0.68–1.63)	0.831
CG+GG	Exposure	61 (19.7)	62 (24.0)	1.41 (0.92–2.16)	0.116
CC	Exposure	21 (6.8)	29 (11.2)	**1.91 (1.04–3.52)** *	**0.037**
rs11614913					
TT	Non-exposure	71 (22.9)	45 (17.4)	1.00 (ref)	—
TC+CC	Non-exposure	157 (50.6)	122 (47.3)	1.23 (0.79–1.91)	0.366
TT	Exposure	26 (8.4)	22 (8.5)	1.34 (0.68–2.63)	0.405
TC+CC	Exposure	56 (18.1)	69 (26.7)	**1.94 (1.16–3.25)** *	**0.011**
rs928508					
AG+GG	Non-exposure	160 (51.6)	123 (47.7)	1.00 (ref)	—
AA	Non-exposure	68 (21.9)	44 (17.1)	0.84 (0.54–1.32)	0.449
AG+GG	Exposure	53 (17.1)	61 (23.6)	1.50 (0.97–2.32)	0.070
AA	Exposure	29 (9.4)	30 (11.6)	1.35 (0.77–2.36)	0.300
rs4919510					
GG	Non-exposure	70 (22.6)	40 (15.5)	1.00 (ref)	—
GC+CC	Non-exposure	158 (51.0)	127 (49.2)	1.41 (0.89–2.21)	0.140
GG	Exposure	26 (8.4)	25 (9.7)	1.68 (0.86–3.30)	0.129
GC+CC	Exposure	56 (18.1)	66 (25.6)	**2.06 (1.22–3.49)** *	**0.007**
rs895819					
TC+CC	Non-exposure	95 (30.6)	78 (30.2)	1.00 (ref)	—
TT	Non-exposure	133 (42.9)	89 (34.5)	0.82 (0.55–1.22)	0.319
TC+CC	Exposure	48 (15.5)	42 (16.3)	1.07 (0.64–1.78)	0.807
TT	Exposure	34 (11.0)	49 (19.0)	**1.76 (1.03–2.98)** *	**0.038**
rs6505162					
CA+AA	Non-exposure	85 (27.4)	57 (22.1)	1.00 (ref)	—
CC	Non-exposure	143 (46.1)	110 (42.6)	1.15 (0.76–1.74)	0.519
CA+AA	Exposure	35 (11.3)	24 (9.3)	1.02 (0.55–1.90)	0.944
CC	Exposure	47 (15.2)	67 (26.0)	**2.13 (1.29–3.51)** *	**0.003**

*: statistically significant results.

Because adenocarcinoma is the most frequent subtype of lung cancer, with a higher incidence in women, we analyzed the interaction between above SNPs in miRNAs and cooking oil fume exposure on lung adenocarcinoma susceptibility in Chinese non-smoking female population (Table 3). For miR-146a rs2910164, miR-196a2 rs11614913, miR-608 rs4919510, miR-27a rs895819 and miR-423 rs6505162 polymorphisms, the risk genotypes combining with the risk factor (cooking oil fume exposure) were significantly associated with lung adenocarcinoma risks, and the corresponding ORs (95%CIs) were 2.13 (1.12–4.04), 2.18 (1.25–3.81), 2.18(1.24–3.83), 2.19 (1.24–3.88) and 2.06 (1.20–3.55). The combination of two risk factors contributed to a higher risk of lung adenocarcinoma than those with only one of them (ORs were 2.13, 1.07 and 1.37 for miR-146a rs2910164, ORs were 2.18, 1.31 and 1.20 for miR-196a2 rs11614913, ORs were 2.18, 1.04 and 1.13 for miR-608 rs4919510, ORs were 2.19, 1.04 and 1.13 for miR-27a rs895819, and ORs were 2.06, 1.13 and 1.13 for miR-423 rs6505162, respectively). In addition, supporting Information S2 Table showed the interaction between SNPs in miRNAs and cooking oil exposure on lung cancer and lung adenocarcinoma susceptibility under other comparisons.

**Table 3:** Interaction between SNPs in miRNAs and cooking oil exposure on lung adenocarcinoma in Chinese non-smoking female population.

SNP	oil	No of controls (%)	No of cases (%)	OR (95%CI)	P value
rs2910164					
CG+GG	Non-exposure	162 (52.3)	87 (44.8)	1.00 (ref)	—
CC	Non-exposure	66 (21.3)	38 (19.6)	1.07 (0.67–1.73)	0.775
CG+GG	Exposure	61 (19.7)	45 (23.2)	1.37 (0.86–2.19)	0.181
CC	Exposure	21 (6.8)	24 (12.4)	**2.13 (1.12–4.04)** *	**0.021**
rs11614913					
TT	Non-exposure	71 (22.9)	32 (16.5)	1.00 (ref)	—
TC+CC	Non-exposure	157 (50.6)	93 (47.9)	1.31 (0.81–2.15)	0.274
TT	Exposure	26 (8.4)	14 (7.2)	1.20 (0.55–2.59)	0.652
TC+CC	Exposure	56 (18.1)	55 (28.4)	**2.18 (1.25–3.81)** *	**0.006**
rs928508					
AG+GG	Non-exposure	160 (51.6)	91 (46.9)	1.00 (ref)	—
AA	Non-exposure	68 (21.9)	34 (17.5)	0.88 (0.54–1.43)	0.603
AG+GG	Exposure	53 (17.1)	45 (23.2)	1.49 (0.93–2.40)	0.097
AA	Exposure	29 (9.4)	24 (12.4)	1.46 (0.80–2.65)	0.220
rs4919510					
GG	Non-exposure	70 (22.6)	31 (16.0)	1.00 (ref)	—
GC+CC	Non-exposure	158 (51.0)	94 (48.5)	1.34 (0.82–2.20)	0.241
GG	Exposure	26 (8.4)	15 (7.7)	1.30 (0.61–2.80)	0.497
GC+CC	Exposure	56 (18.1)	54 (27.8)	**2.18(1.24–3.83)** *	**0.007**
rs895819					
TC+CC	Non-exposure	95 (30.6)	51 (26.3)	1.00 (ref)	—
TT	Non-exposure	133 (42.9)	74 (38.1)	1.04 (0.67–1.62)	0.874
TC+CC	Exposure	48 (15.5)	29 (14.9)	1.13 (0.64–2.00)	0.686
TT	Exposure	34 (11.0)	40 (20.6)	**2.19 (1.24–3.88)** *	**0.007**
rs6505162					
CA+AA	Non-exposure	85 (27.4)	43 (22.2)	1.00 (ref)	—
CC	Non-exposure	143 (46.1)	82 (42.3)	1.13 (0.72–1.79)	0.519
CA+AA	Exposure	35 (11.3)	20 (10.3)	1.13 (0.58–2.19)	0.718
CC	Exposure	47 (15.2)	49 (25.3)	**2.06 (1.20–3.55)** *	**0.009**

*: statistically significant results.

Table 4 showed the quantitative and statistically significant analyses results about the measures of biological interaction between SNPs in miRNAs and cooking oil exposure on lung cancer and adenocarcinoma in Chinese non-smoking female population. There are three measures of interaction and their confidence intervals. If there is no biological interaction, RERI and AP are equal to 0 and S is equal to 1. All the measures of biological interaction indicate that there were not indeed biological interactions between the six SNPs of miRNAs and exposure to cooking oil fumes on an additive scale. Logistic models suggested that the gene-environment interactions were not statistically significant on a multiplicative scale (data not shown). From the results of our analyses, there may be no statistically significant interaction between six miRNA SNPs and cooking oil fume exposure in lung cancer or lung adenocarcinoma among nonsmoking females in China.

**Table 4:** Interaction measures between SNPs in miRNAs and cooking oil exposure on lung cancer and adenocarcinoma in Chinese non-smoking female population.

SNP	Lung cancer			Lung adenocarcinoma		
	Measure	Estimate	95%CI	Measure	Estimate	95%CI
rs2910164						
	RERI	0.456	-0.796, 1.707	RERI	0.682	-0.740, 2.105
	AP	0.238	-0.315, 0.792	AP	0.321	-0.204, 0.846
	S	1.999	0.277, 14.402	S	2.531	0.308, 20.768
rs11614913						
	RERI	0.383	-0.704, 1.470	RERI	0.670	-0.510, 1.850
	AP	0.197	-0.343, 0.737	AP	0.308	-0.196, 0.811
	S	1.683	0.259, 10.910	S	2.317	0.254, 21.148
rs928508						
	RERI	0.007	-0.958, 0.972	RERI	0.083	-0.993, 1.159
	AP	0.005	-0.711, 0.721	AP	0.057	-0.664, 0.778
	S	1.020	0.061, 17.197	S	1.223	0.084, 17.735
rs4919510						
	RERI	-0.027	-1.315, 1.262	RERI	0.531	-0.695, 1.758
	AP	-0.013	-0.639, 0.613	AP	0.244	-0.292, 0.780
	S	0.975	0.298, 3.196	S	1.822	0.296, 11.219
rs895819						
	RERI	0.875	-0.039, 1.788	RERI	1.030	-0.137, 2.196
	AP	0.498	0.103, 0.893	AP	0.470	0.074, 0.866
	S	-6.332	—	S	7.363	0.032, 1707.376
rs6505162						
	RERI	0.956	-0.040, 1.952	RERI	0.798	-0.282, 1.877
	AP	0.450	0.066, 0.834	AP	0.387	-0.065, 0.840
	S	6.636	0.039, 1141.808	S	4.032	0.102, 159.023

RERI: relative excess risk due to interaction, AP: attributable proportion due to interaction, S: synergy index, 95%CI: 95% confidence interval.

—: The results cannot be calculated.

## Discussion

In recent years, the etiological study of lung cancer remains popular all over the world, but the results are inconsistent. It is well known that besides tobacco smoking, other impact factors of lung cancer are not definitive. Cigarette smoking cannot fully explain the epidemiologic characteristics of lung cancer in Chinese women, who smoke rarely but have lung cancer relatively often. Undoubtedly non-smoking females are the ideal subjects to examine unknown, yet important environmental and genetic factors of lung cancer. Our research team has been going on the study of risk factors and genetic susceptibility of lung cancer in Chinese population. In previous studies, we reported the associations between cooking oil fume exposure and lung cancer risks. More recently, we analyze the miRNA SNPs and lung cancer risks. Considering the important role of gene-environment interaction in the development of lung cancer, in the present study we evaluated the interaction between six functional polymorphisms of six miRNAs and lung cancer risks in a Chinese non-smoking female population.

To the best of our knowledge, this is the first study comprehensively exploring the interaction between miRNA SNPs and the environmental risk factor on both additive scale and multiplicative scale by qualitative and quantitative analyses. The qualitative results by cross-over analyses suggested that there may be interaction between six miRNA SNPs and cooking oil fume exposure in the development of lung cancer or lung adenocarcinoma among Chinese non-smoking female population. However, further quantitative analyses on both additive scale and multiplicative scale showed that no statistically significant interaction between miRNA SNPs and cooking oil fume exposure were found in lung cancer or lung adenocarcinoma among nonsmoking females in China.

In the present study, individuals with exposure to cooking oil fume had a 1.52-fold increased risk to develop lung cancer, which was concordant with our previous reports [14–18]. There are studies showing that cooking oil fume condensates can induce DNA damages and influence DNA cross-links in a certain concentration [24–25]. Tung et al found that exposure to cooking oil fume could inhibit cell growth and induce oxidative stress in lung epithelial cells [26]. There are other studies suggesting the important roles of cooking oil fume exposure in lung cancer risk among nonsmoking women [27–29]. Above evidences support the important roles of cooking oil fume exposure in lung cancer development.

A strong link between altered miRNAs, either structure changes or expression of mature miRNA, and cancer risk has been established, opening up a new avenue of investigation for molecular mechanisms of cancer development [30]. Although their biological functions remain largely unclear, there are studies demonstrating that miRNAs may function as oncogenes and/or tumor suppressor genes [30–34]. SNPs or mutations of miRNAs may affect their property through altering miRNA expression and/or maturation, and thus may contribute to cancer risk [9,35]. Researchers forecast that miRNAs could become ideal candidates for cancer predisposition factor because their small variation may affect thousands of target mRNAs and result in diverse functional consequences. However, the role of genetic variants in miRNAs in cancer susceptibility is mostly unknown.

Most of the etiological studies of cancer did not analyze the gene-environment interaction, which is considered more important in the etiology of cancer, or only analyzed the multiplicative interaction by adding the interaction term in logistic regression. An editorial have stated that statistical interaction is quite different from biological interaction, the former may reflect either departure from additivity or multiplicativity depending on the chosen statistical model and the degree of biological interaction between risk factors is measured as the deviation from additivity and not as deviation from multiplicativity [36]. The logistic regression model is probably the most commonly used statistical model in epidemiologic analysis to day, however logistic regression is inherently multiplicative, so it is widely used to only analyze multiplicative interaction. Thus, a significant interaction term in such a model implies a multiplicative relation between the disease and the presence of an interaction term implies departure from multiplicativity, rather than from additivity. Therefore, the interaction term in logistic model has no direct ability to state whether or not biological interaction is present. On the other hand, the biological interaction can still be explored from the results of a logistic regression model, but this requires that the model is defined in a special way and that the analysis is done adequately. Andersson et al. describe how a logistic regression model can be defined in order to produce the output that is needed for assessment of biological interaction [23].

In present study, the interaction analysis results about comparing ORs of different gene-environment combination by cross-over analyses showed that in five miRNA SNPs the combination of the risk genotypes with risk factor (cooking oil fume exposure) contributed to a significantly higher risk of lung cancer, suggesting there may be gene-environment interaction. However, further statistical analyses for additive interaction and multiplicative interaction showed that interactions between miRNA SNPs and cooking oil fume exposure were not significant, either from multiplicativity using interaction term of logistic regression or from additivity by Andersson et al.’s method. The possible reason is that the sample size of present study is too small to obtain the statistically significant results. So larger population of lung cancer is needed to verify the conclusion in future. Because it is just a statistical estimation, further studies concerning their biological validity are required.

This study is the first study to evaluate the gene-environment interaction between miRNAs SNPs and environmental risk factors in the development of lung cancer on both multiplicative and additive scale. Although the results were statistically significant, the idea and method to comprehensively assess interaction between two factors are valuable and have indicative significance for future similar studies. The most important limitations of study were the small sample size and absence of other factors including potential confounding factors in the analyses. Consequently, future studies with larger sample size population of lung cancer, considering more impact factors and concerning their biological validity are required to verify the conclusion.

In summary, this study firstly reported the interaction of single nucleotide polymorphisms in miRNAs and the environmental risk factor in the development of lung cancer, stating there were no statistically significant interaction. Our study would have a certain positive significance in the understanding of lung cancer pathogenesis and in the future analysis of interaction.

## Conclusions

The interactions between miRNA SNPs and cooking oil fume exposure suggested by ORs of different combination were not statistically significant.

## Supporting Information

S1 Table Combination of SNPs in miRNAs and cooking oil exposure on susceptibility of lung cancer and lung adenocarcinoma in Chinese non-smoking female population under other comparisons. (DOC)

S2 Table Interaction between SNPs in miRNAs and cooking oil exposure on lung cancer susceptibility in Chinese non-smoking female population under other comparisons. (DOC)
