# Supplementary material for: Interaction between Polymorphisms in Pre-MiRNA Genes and Cooking Oil Fume Exposure on the Risk of Lung Cancer in Chinese Non-Smoking Female Population
Source: PLoS One. 2015 Jun 17;10(6):e0128572. doi: 10.1371/journal.pone.0128572 (PMC4471348; doi:10.1371/journal.pone.0128572)
Supplement: S1 Table — (DOC) [file pone.0128572.s001.doc]

**S1 Table**

**Combination of SNPs in miRNAs and cooking oil exposure on susceptibility of lung cancer and lung adenocarcinoma in Chinese non-smoking female population under other comparisons**

|  | lung cancer | |  | | lung adenocarcinoma | |
| --- | --- | --- | --- | --- | --- | --- |
|  | OR [95%CI] | P value |  | OR [95%CI] | | P value |
| rs2910164 |  |  |  |  | |  |
| GG+Non-exposure vs CC+Exposure | 0.49 (0.24-1.02) | 0.055 |  | 0.47(0.22-1.02) | | 0.055 |
| CG+Non-exposure vs CC+Exposure | 0.54(0.29-1.00) | 0.050 |  | 0.47(0.24-0.91) | | 0.025 |
| CC+Non-exposure vs CC+Exposure | 0.55(0.28-1.07) | 0.080 |  | 0.50(0.25-1.02) | | 0.058 |
| GG+Exposure vs CC+Exposure | 0.58(0.24-1.38) | 0.216 |  | 0.53(0.21-1.32) | | 0.172 |
| CG+Exposure vs CC+Exposure | 0.81(0.40-1.64) | 0.562 |  | 0.70(0.34-1.48) | | 0.356 |
| GG+Non-exposure vs CG+Non-exposure | 0.91(0.53-1.57) | 0.741 |  | 1.00(0.55-1.80) | | 0.986 |
| CG+Non-exposure vs CC+Non-exposure | 0.98(0.62-1.55) | 0.918 |  | 0.93(0.57-1.54) | | 0.790 |
| CC+Non-exposure vs GG+Exposure | 0.95(0.45-2.01) | 0.887 |  | 0.96(0.42-2.18) | | 0.921 |
| GG+Exposure vs CG+Exposure | 0.71(0.33-1.56) | 0.396 |  | 0.75(0.32-1.74) | | 0.498 |
|  |  |  |  |  | |  |
| rs11614913 |  |  |  |  | |  |
| TT+Non-exposure vs CC+Exposure | 0.60(0.29-1.27) | 0.180 |  | 0.62(0.27-1.43) | | 0.263 |
| TC+Non-exposure vs CC+Exposure | 0.77(0.38-1.55) | 0.465 |  | 0.85(0.39-1.85) | | 0.687 |
| CC+Non-exposure vs CC+Exposure | 0.65(0.30-1.44) | 0.290 |  | 0.74(0.31-1.76) | | 0.494 |
| TT+Exposure vs CC+Exposure | 0.80(0.34-1.89) | 0.614 |  | 0.75(0.28-1.96) | | 0.551 |
| TC+Exposure vs CC+Exposure | 1.25(0.58-2.69) | 0.575 |  | 1.53(0.66-3.54) | | 0.319 |
| TT+Non-exposure vs TC+Non-exposure | 0.78(0.49-1.24) | 0.295 |  | 0.73(0.44-1.22) | | 0.233 |
| TC+Non-exposure vs CC+Non-exposure | 1.18(0.69-2.01) | 0.545 |  | 1.16(0.65-2.06) | | 0.626 |
| CC+Non-exposure vs TT+Exposure | 0.81(0.39-1.69) | 0.580 |  | 0.99(0.44-2.24) | | 0.982 |
| TT+Exposure vs TC+Exposure | 0.64(0.32-1.30) | 0.221 |  | 0.49(0.22-1.07) | | 0.072 |
|  |  |  |  |  | |  |
| rs928508 |  |  |  |  | |  |
| GG+Non-exposure vs AA+Exposure | 0.84(0.44-1.63) | 0.613 |  | 0.82(0.41-1.66) | | 0.586 |
| AG+Non-exposure vs AA+Exposure | 0.70(0.39-1.26) | 0.234 |  | 0.63(0.34-1.18) | | 0.149 |
| AA+Non-exposure vs AA+Exposure | 0.63(0.33-1.18) | 0.148 |  | 0.60(0.31-1.19) | | 0.146 |
| GG+Exposure vs AA+Exposure | 1.29(0.52-3.19) | 0.583 |  | 1.41(0.55-3.62) | | 0.475 |
| AG+Exposure vs AA+Exposure | 1.06(0.55-2.06) | 0.861 |  | 0.91(0.45-1.87) | | 0.804 |
| GG+Non-exposure vs AG+Non-exposure | 1.20(0.73-1.99) | 0.476 |  | 1.30(0.75-2.26) | | 0.343 |
| AG+Non-exposure vs AA+Non-exposure | 1.12(0.70-1.80) | 0.635 |  | 1.04(0.62-1.75) | | 0.870 |
| AA+Non-exposure vs GG+Exposure | 0.49(0.21-1.12) | 0.091 |  | 0.43(0.18-1.03) | | 0.057 |
| GG+Exposure vs AG+Exposure | 1.22(0.51-2.87) | 0.657 |  | 1.54(0.63-3.80) | | 0.346 |
|  |  |  |  |  | |  |
| rs4919510 |  |  |  |  | |  |
| GG+Non-exposure vs CC+Exposure | 0.60(0.30-1.21) | 0.150 |  | 0.57(0.27-1.20) | | 0.136 |
| GC+Non-exposure vs CC+Exposure | 0.84(0.44-1.61) | 0.604 |  | 0.74(0.37-1.47) | | 0.390 |
| CC+Non-exposure vs CC+Exposure | 0.83(0.39-1.76) | 0.629 |  | 0.82(0.37-1.81) | | 0.623 |
| GG+Exposure vs CC+Exposure | 1.01(0.45-2.24) | 0.990 |  | 0.74(0.30-1.79) | | 0.500 |
| GC+Exposure vs CC+Exposure | 1.39(0.67-2.92) | 0.378 |  | 1.39(0.64-3.03) | | 0.402 |
| GG+Non-exposure vs GC+Non-exposure | 0.71(0.44-1.14) | 0.153 |  | 0.76(0.46-1.28) | | 0.306 |
| GC+Non-exposure vs CC+Non-exposure | 1.02(0.59-1.75) | 0.957 |  | 0.91(0.51-1.62) | | 0.736 |
| CC+Non-exposure vs GG+Exposure | 0.83(0.40-1.71) | 0.606 |  | 1.11(0.49-2.50) | | 0.790 |
| GG+Exposure vs GC+Exposure | 0.72(0.35-1.47) | 0.367 |  | 0.53(0.24-1.17) | | 0.115 |
|  |  |  |  |  | |  |
| rs895819 |  |  |  |  | |  |
| CC+Non-exposure vs TT+Exposure | 0.93(0.35-2.44) | 0.875 |  | 0.85(0.30-2.38) | | 0.757 |
| TC+Non-exposure vs TT+Exposure | 0.53(0.31-0.92) | 0.023 |  | 0.42(0.23-0.75) | | 0.003 |
| TT+Non-exposure vs TT+Exposure | 0.46(0.28-0.78) | 0.003 |  | 0.47(0.28-0.81) | | 0.006 |
| CC+Exposure vs TT+Exposure | 0.39(0.12-1.25) | 0.113 |  | 0.38(0.11-1.34) | | 0.131 |
| TC+Exposure vs TT+Exposure | 0.66(0.35-1.23) | 0.192 |  | 0.55(0.28-1.08) | | 0.080 |
| CC+Non-exposure vs TC+Non-exposure | 1.74(0.69-4.37) | 0.240 |  | 2.05(0.76-5.54) | | 0.158 |
| TC+Non-exposure vs TT+Non-exposure | 1.15(0.76-1.74) | 0.521 |  | 0.88(0.55-1.40) | | 0.583 |
| TT+Non-exposure vs CC+Exposure | 1.21(0.39-3.71) | 0.746 |  | 1.25(0.37-4.21) | | 0.716 |
| CC+Exposure vs TC+Exposure | 0.59(0.18-1.91) | 0.375 |  | 0.69(0.19-2.50) | | 0.575 |
|  |  |  |  |  | |  |
| rs6505162 |  |  |  |  | |  |
| AA+Non-exposure vs CC+Exposure | 0.32(0.10-0.98) | 0.046 |  | 0.35(0.10-1.17) | | 0.089 |
| CA+Non-exposure vs CC+Exposure | 0.49(0.30-0.83) | 0.007 |  | 0.51(0.29-0.88) | | 0.016 |
| CC+Non-exposure vs CC+Exposure | 0.54(0.35-0.85) | 0.007 |  | 0.55(0.34-0.89) | | 0.015 |
| AA+Exposure vs CC+Exposure | 0.47(0.08-2.91) | 0.415 |  | 0.64(0.10-4.00) | | 0.633 |
| CA+Exposure vs CC+Exposure | 0.48(0.25-0.93) | 0.030 |  | 0.54(0.27-1.09) | | 0.085 |
| AA+Non-exposure vs CA+Non-exposure | 0.65(0.21-1.97) | 0.444 |  | 0.69(0.21-2.31) | | 0.547 |
| CA+Non-exposure vs CC+Non-exposure | 0.91(0.59-1.41) | 0.682 |  | 0.92(0.57-1.48) | | 0.727 |
| CC+Non-exposure vs AA+Exposure | 1.15(0.19-7.03) | 0.877 |  | 0.86(0.14-5.25) | | 0.870 |
| AA+Exposure vs CA+Exposure | 0.97(0.15-6.29) | 0.974 |  | 1.19(0.18-7.77) | | 0.859 |
