# Supplementary material for: Interaction between Polymorphisms in Pre-MiRNA Genes and Cooking Oil Fume Exposure on the Risk of Lung Cancer in Chinese Non-Smoking Female Population
Source: PLoS One. 2015 Jun 17;10(6):e0128572. doi: 10.1371/journal.pone.0128572 (PMC4471348; doi:10.1371/journal.pone.0128572)
Supplement: S2 Table — (DOC) [file pone.0128572.s002.doc]

**S2 Table**

**Interaction between SNPs in miRNAs and cooking oil exposure on lung cancer susceptibility in Chinese non-smoking female population under other comparisons**

|  | lung cancer | |  | | lung adenocarcinoma | |
| --- | --- | --- | --- | --- | --- | --- |
|  | OR [95%CI] | P value |  | OR [95%CI] | | P value |
| rs2910164 |  |  |  |  | |  |
| CG/GG+Non-exposure vs CC+Exposure | 0.52(0.28-0.96) | 0.037 |  | 0.47(0.25-0.89) | | 0.021 |
| CC+Non-exposure vs CC+Exposure | 0.55(0.28-1.07) | 0.080 |  | 0.50(0.25-1.02) | | 0.058 |
| CG/GG+Exposure vs CC+Exposure | 0.74(0.38-1.43) | 0.365 |  | 0.65(0.32-1.30) | | 0.221 |
| CG/GG+Non-exposure vs CC+Non-exposure | 0.95(0.62-1.48) | 0.831 |  | 0.93(0.58-1.50) | | 0.775 |
| CC+Non-exposure vs CG/GG+Exposure | 0.75(0.45-1.24) | 0.259 |  | 0.78(0.45-1.36) | | 0.381 |
|  |  |  |  |  | |  |
| rs11614913 |  |  |  |  | |  |
| TT+Non-exposure vs TC/CC+Exposure | 0.51(0.31-0.86) | 0.011 |  | 0.46(0.26-0.80) | | 0.006 |
| TC/CC+Non-exposure vs TC/CC+Exposure | 0.63(0.41-0.96) | 0.033 |  | 0.60(0.38-0.95) | | 0.028 |
| TT+Exposure vs TC/CC+Exposure | 0.69(0.35-1.34) | 0.270 |  | 0.55(0.26-1.16) | | 0.116 |
| TT+Non-exposure vs TC/CC+Non-exposure | 0.82(0.52-1.27) | 0.366 |  | 0.76(0.47-1.24) | | 0.274 |
| TC/CC+Non-exposure vs TT+Exposure | 0.92(0.50-1.70) | 0.786 |  | 1.10(0.55-2.21) | | 0.789 |
|  |  |  |  |  | |  |
| rs928508 |  |  |  |  | |  |
| AG/GG+Non-exposure vs AA+Exposure | 0.74(0.42-1.30) | 0.300 |  | 0.69(0.38-1.25) | | 0.220 |
| AA+Non-exposure vs AA+Exposure | 0.63(0.33-1.18) | 0.148 |  | 0.60(0.31-1.19) | | 0.146 |
| AG/GG+Exposure vs AA+Exposure | 1.11(0.59-2.09) | 0.740 |  | 1.03(0.52-2.01) | | 0.940 |
| AG/GG+Non-exposure vs AA+Non-exposure | 1.19(0.76-1.86) | 0.449 |  | 1.14(0.70-1.85) | | 0.603 |
| AA+Non-exposure vs AG/GG+Exposure | 0.56(0.33-0.95) | 0.033 |  | 0.59(0.33-1.04) | | 0.070 |
|  |  |  |  |  | |  |
| rs4919510 |  |  |  |  | |  |
| GG+Non-exposure vs GC/CC+Exposure | 0.49(0.29-0.82) | 0.007 |  | 0.46(0.26-0.81) | | 0.007 |
| GC/CC+Non-exposure vs GC/CC+Exposure | 0.68(0.45-1.04) | 0.078 |  | 0.62(0.39-0.97) | | 0.037 |
| GG+Exposure vs GC/CC+Exposure | 0.82(0.42-1.57) | 0.542 |  | 0.60(0.29-1.25) | | 0.172 |
| GG+Non-exposure vs GC/CC+Non-exposure | 0.71(0.45-1.12) | 0.140 |  | 0.74(0.45-1.22) | | 0.241 |
| GC/CC+Non-exposure vs GG+Exposure | 0.84(0.46-1.52) | 0.556 |  | 1.03(0.52-2.05) | | 0.930 |
|  |  |  |  |  | |  |
| rs895819 |  |  |  |  | |  |
| TC/CC+Non-exposure vs TT+Exposure | 0.57(0.34-0.97) | 0.038 |  | 0.46(0.26-0.81) | | 0.007 |
| TT+Non-exposure vs TT+Exposure | 0.46(0.28-0.78) | 0.003 |  | 0.47(0.28-0.81) | | 0.006 |
| TC/CC+Exposure vs TT+Exposure | 0.61(0.33-1.11) | 0.104 |  | 0.51(0.27-0.98) | | 0.044 |
| TC/CC+Non-exposure vs TT+Non-exposure | 1.23(0.82-1.83) | 0.319 |  | 0.97(0.62-1.50) | | 0.874 |
| TT+Non-exposure vs TC/CC+Exposure | 0.77(0.47-1.25) | 0.287 |  | 0.92(0.54-1.58) | | 0.766 |
|  |  |  |  |  | |  |
| rs6505162 |  |  |  |  | |  |
| CA/AA+Non-exposure vs CC+Exposure | 0.47(0.29-0.78) | 0.003 |  | 0.49(0.28-0.84) | | 0.009 |
| CC+Non-exposure vs CC+Exposure | 0.54(0.35-0.85) | 0.007 |  | 0.55(0.34-0.89) | | 0.015 |
| CA/AA+Exposure vs CC+Exposure | 0.48(0.25-0.91) | 0.025 |  | 0.55(0.28-1.08) | | 0.083 |
| CA/AA+Non-exposure vs CC+Non-exposure | 0.87(0.57-1.32) | 0.519 |  | 0.88(0.56-1.39) | | 0.590 |
| CC+Non-exposure vs CA/AA+Exposure | 1.12(0.63-2.00) | 0.696 |  | 1.00(0.54-1.85) | | 0.991 |
